# Common Difficulties in Forceps Delivery

**Published:** 1910-06-04

**Authors:** T. G. Stevens

**Affiliations:** A Lecture at the Polyclynic.


### Hospital Clinics.

### COMMON DIFFICULTIES IN FORCEPS DELIVERY.

By T. G. STEVENS, M.D., F.R.C.S., A Lecture at the Polyclynic.

This is, of course, an exceedingly interesting subject, and one which is always before us. It leads to considerable troubles in domestic life, and to much after-suffering on the part of the patient. If I were asked what is the commonest and most difficult complication in midwifery, I should say occipito-posterior positions; and I suppose by far the greater number of obstetric tragedies—for one can scarcely call them anything else—occur as the result of the treatment of occipito-posterior positions of the fœtus.

I propose to say a little about forceps extractions in occipito-posterior positions of the fœtus. You are familiar with the lingering labour which occurs in the majority of these cases, and also with the way in which an occipito-posterior position of the fœtus will gradually, under the influence of uterine contractions, rotate without extraneous aid. But you also know how long this takes, and how often the occiput does not rotate to the anterior part of the pelvis as it should do, but remains posteriorly, and in the end rotates into the hollow of the sacrum, leading to persistent occipito-posterior delivery.

Delivery of the fœtus with the occiput directed backwards is a calamity for a primipara, because it nearly always leads to very severe lacerations, and often to the death of the fœtus from compression injuries. It may not mean so serious a trouble for the multipara, whose soft parts have been dilated and will stretch more readily. In a primipara, occipito-posterior positions are often more difficult than the mere fact of being a primipara would warrant; sometimes because the child is small. Small children do not rotate easily when the occiput is posterior. The large head encounters more resistance, and therefore is much more likely to rotate than the small head, which will enter the pelvis in whatever position it happens to find itself. Therefore the plea must be on behalf of the primipara with the occipito-posterior position of the fœtus. It is not out of place for us to consider why these positions are so difficult. The common occipito-posterior position is the one with the occiput pointing to the right. That with the occiput pointing to the left is rarer. It is stated in the text-books to be 2 per cent., but that is very low. I think from 6 per cent. to 8 per cent. is nearer; whereas occipito-posterior positions to the right number about 30 per cent. of all vertex presentations.

### Lingering Labour.

Why is it that these cases give rise to lingering labours? This is a point which is not sufficiently brought out in the text-books. They tell you the reason for the prolonged labour is the want of flexion, whereby the occiput is not low enough in its descent in the pelvis to reach the pelvic floor and thereby to rotate to the front. I may recall the law which governs internal rotation; it is that whatever portion of the fœtal head touches the pelvic floor first, that part will turn to the front. In a right occipito-posterior presentation one of the chief things that we first notice is that the head, instead of descending in that position, descends midway between flexion and extension, opposing a longer diameter, the occipito-frontal, to the pelvic plane which it happens to lie in, and also opposing a much larger circumference of the skull than that which surrounds the suboccipito-bregmatic diameter. But that is not everything; it is not merely because the head is extended and because a larger circumference lies across the pelvis that the difficulty arises, but for another reason—namely, that the head descending in the position of the right occipito-posterior partly extended with regard to the pelvis will go down as far as the pelvic floor. I mean particularly the levator ani muscles and the fasciæ. These muscles and fasciæ form a kind of sling with sloping sides and with an elliptical opening in front, and these are the factors which cause internal rotation. When the occiput hits one side it turns to the front; if the forehead hits the other side first, it turns to the front. But if the head is midway between flexion and extension, and drops to the pelvic floor, which side hits the pelvic floor first? Neither side. And this is the reason these cases are difficult: both sides try to turn to the front, which is in accordance with the law, but neither of them does. The head descends to a certain position in the pelvis, and there it remains. The uterine contractions go on all the time, and practically the result of the case will depend on the strength of the uterine contractions. So much so that some authorities believe that if the uterine contractions can be strengthened, every occipito-posterior case will end by normal rotation. That is a desideratum which is too much to hope for. We know that an elderly primipara, or a multipara who has had many children, has weak uterine contractions from the start, and whatever you do you cannot strengthen them. So that the giving of sedatives to these women in the hope that the contractions will be more powerful when the patient awakes is not universally applicable. It may answer in cases where the pains have been strong and have become weak, but it will not answer in a majority of cases. So we have the position in which the head has extended to a certain degree, the uterus acting regularly but not very strongly, and the result is a deadlock. It is an impacted vertex presentation in the sense that the powers exerted above are not sufficient to expel the head. The forceps are, as a rule, applied at this stage, the head lying a little obliquely perhaps. It is in the worst position for forceps extraction. One blade will go over the malar diameter and the other behind the ear, and a strong pull will not move the head because both sides are touching the pelvic floor. To get easy forceps extraction the head must be in the antero-posterior diameter. What is the best way of dealing with it? As it is a fact that the want of flexion of the head has brought about this condition from the start, the advice is to flex the head in the hope that the uterine contractions will bring about rotation. This flexion of the head is to be brought about by pulling on the occiput from below with your fingers on it, a very imperfect mechanical advantage; or by pushing up the forehead from within in the same way. We cannot do the two together, because we cannot put both our hands into the vagina at once. If the head can be flexed by the fingers, or by some such instrument as the vectis, which is obsolete but could be used for this purpose, and the uterus acted strongly enough to drive it down, rotation will occur.

### Rotation of the Head.

If this proves ineffectual the next manœuvre recommended is to rotate the head artificially with the hand. It is impossible as a rule to rotate the head with two fingers; you must grasp it with the whole hand, and therefore the whole hand must be introduced so as to rotate the head. When you let go the head it will go back while you are putting on one blade of the forceps. The reason is that you have not rotated the body of the child, and it is very difficult to do so from the abdomen, as we are mostly recommended to do. We are told to pull the shoulder across the middle line so as to rotate the body, which is well if you can do it; but as a rule you cannot feel the anterior shoulder, and if you can you are working at a mechanical disadvantage through the abdominal walls to bring it round. The best thing to do is to put the whole hand into the vagina and pass it above the head; and it is surprising how easy it is to do; and get hold of the shoulder from within, leaving the head against your hand while turning the shoulder to carry the head round with your hand. But you must be able to reach the shoulder. The hands of some of us are not made for these manipulations. There is no doubt the man with a big hand or a fat hand and short fingers cannot carry out this manœuvre very satisfactorily.

Of course this cannot be done without an anæsthetic, and I know that in general practice a great difficulty arises because we so often have to do everything ourselves: give the anæsthetic, put on the forceps, and rotate the head, while somebody helps to keep the patient under the anæsthetic. When you get a difficult occipito-posterior case it is far more important to you and to the mother than a case of contracted pelvis of the third degree, because the contracted pelvis has to be operated upon, whereas you are expected to be able to terminate the former case successfully for the child and for the mother.

Another practical point is that when the head lies in the pelvis in the third position or is rotated backwards into the hollow of the sacrum, it is almost impossible for you to tell by examination whether the head was originally right occipito-posterior or left, and you may find that in rotating the head you sometimes have to turn it round the right side of the pelvis, and sometimes it is much more easily turned to the left side. So if it will not turn one way it may another.

Another small manœuvre which is of great use in some of these cases is to put on the upper blade of the forceps first. With the patient lying with the occiput pointing to the right sacro-iliac synchondrosis it tends to go back again. But the application of the upper blade will give you a leverage on the head and enable you to keep it in the antero-posterior position. It is difficult to put the blade on because you have to reverse the way in which the handles lock. When the head has been rotated and lies in the long diameter of the outlet there is no difficulty in delivering it. In many of these cases, were it not for the fact that the patient is under an anæsthetic and would like to wake up delivered, there is no reason why she should not be left to deliver herself, with great advantage to both the child and herself. But when the patient is once under the anæsthetic she hopes that when she comes round everything will be finished. Therefore it is usual to terminate delivery by forceps. This will be a simple forceps extraction if you have rotated the head in the manner I suggest.

### A Question of Diagnosis.

Sometimes difficulties arise because we cannot tell whether the case is one of occipito-posterior presentation or not. It is not easy to distinguish these cases in some women. It has to be done by a combined external and internal examination. External abdominal examination was not taught a few years ago as it is now; we are in the habit now of trying to make up our minds by external examination alone what the position of the child really is, and it is astonishing how much information you can get if you are in the habit of carefully palpating every case. It is no exaggeration to say that we ought to be able to diagnose every position and presentation of the child by abdominal palpation only; and it is a tremendous advantage to be able to do so, because then, whatever vaginal examination has to be made, it can be made merely for the purpose of distinguishing the condition of the cervix and the soft parts without respect to the position and presentation of the child. But if you have a difficult case in which labour is prolonged, in which the advance of the head is slow, make certain whether the case is an occipito-posterior one. The only way to do it with certainty with the woman in labour is to introduce the whole hand, to grasp the head, to feel the position of the ears, and in that way to make certain. There should not be any difficulty in introducing the whole hand, and there should not be any danger, and it is of the greatest possible value in difficult cases of this sort.

Now let us consider what other difficult forceps extractions there may be. There are not many, and the more skilful you become in obstetric manipulation the fewer difficult forceps cases there will be. The more carefully cases are investigated beforehand, the fewer cases will have to be delivered by forceps under difficulties. Take, for instance, cases of minor contractions of the pelvis, cases in which, perhaps, the true conjugate diameter measures $3\frac{3}{4}$ inches, or even a little less. These are the cases in which the head is very liable to be delayed at the brim. In these moderate degrees of pelvic contraction the head may be just in the brim, but it will be more or less movable in an upwards or sideways direction. And it is in these cases that the temptation to put on forceps early is very great, and that, perhaps, injuries to the mother and child are liable to occur. These are the cases which will always afford difficult forceps extractions under certain circumstances. The head will have an obliquity towards one or other shoulder at the brim. The reason of this obliquity is that the sub-parietal supra-parietal diameter is a smaller diameter than the bi-parietal, and Nature attempts by this oblique position to bring the head to the brim and allow it to get through in that way. The question which has to be decided in all these cases of mild pelvic contraction is whether the head will by any means come through with the child alive? And how will you make up your mind to that? You examine the patient, and find the head above the brim. It may be showing a slight attempt by becoming fixed in the brim. How shall we determine whether it will come through or not? There is no doubt that it is not right to put on forceps as a tentative measure. You sometimes hear a man say, "I had a pull to see if it would come into the brim, but I had to give it up." That is not good midwifery; you should be able to decide whether the head will go into the brim before you put on the forceps, and if you think it will not, the case must be treated in some other way. You may try whether you can push the head down from above, and you can only do it satisfactorily under an anæsthetic. If you can push the head into the brim, you can leave the case to Nature as long as possible. Sometimes bimanual examination is very useful; but with the whole hand in the vagina and the other hand above the pubes you can palpate the head carefully and see how much room there is in the transverse diameter. If there is an inch of room here there is hope that the narrow portion may pass through the brim with extension movement; but if there is no room at the sides it is obvious that the head, having arrived at a certain position, it will stick if the diameters of pelvis and head bear a certain relation to one another. Therefore the result of your method of examination must tell you whether the head will go into the brim or not.

### Version *versus* Forceps.

The next point is: having decided whether the head has a chance of being delivered, what is the best thing to do? The best thing I can tell you to do is to leave it alone. I mean you must watch the case carefully to see the first signs of the mother getting exhausted, or for signs of the fœtal heart failing or getting embarrassed. Extreme rapidity of the fœtal heart and extreme slowness both indicate embarrassment and impending death. An increase in the rate of the mother's pulse and a rise in temperature indicate exhaustion on her part. The reason for leaving these cases as long as possible is to allow the head to become moulded as much as it can at the brim. A head which could not be delivered by forceps one hour, because no moulding has occurred, will be delivered easily three hours hence, the patient being still in good condition, on account of the moulding which has occurred in the meantime. Moulding must always be extreme in those cases which are left either to Nature or to forceps deliveries with contracted pelvis. You may say, Why discuss forceps in connection with flat pelvis, when it has been the classical treatment to recommend versions for the minor degrees of contraction of the pelvis? There is no doubt that the question of prophylactic version *versus* forceps can be discussed to almost any extent, and yet no conclusion may be come to. And I think the reason is that where one man is exceedingly skilful in delivering with version, perhaps seven or eight will be skilful in delivering by forceps. And the forceps will always get a live child through a slightly contracted pelvis more readily than will version, because of the extreme difficulty in some of these cases of bringing down the arms and dealing with the after-coming head, which is liable to become extended. There is no need for me to discuss prophylactic version. It has been practically abandoned at the Rotunda in Dublin, and in London, and is seldom done for flat pelvis. If you consider general contraction of the pelvis, prophylactic version has no place at all; forceps is the only treatment for that, and is useless there unless moulding has been allowed to take place.

Now, with regard to difficult forceps extraction—because in every one of these cases of slight pelvic contraction the forceps will generally have to be put on with the head high up at the brim—it is not so much the difficulty of putting the forceps on or of getting a firm grip, but of pulling in the right direction; and here I shall lead the way to an advocacy of the axis-traction forceps. Axis-traction forceps are not so useful for ordinary forceps extraction with the head lying in the axis of the pelvic floor low down. The great writers on the subject of axis-traction forceps say that even there delivery is brought about by much less force than it is with ordinary forceps. But still, we are so familiar with the practical use of the forceps with the head low down that there is no practical utility in discussing axis-traction forceps under these circumstances.

